# Supplementary material for: Water induced sediment levitation enhances downslope transport on Mars
Source: Nat Commun. 2017 Oct 27;8:1151. doi: 10.1038/s41467-017-01213-z (PMC5658360; doi:10.1038/s41467-017-01213-z)
Supplement: Supplementary file 1 — Supplementary Information [file 41467_2017_1213_MOESM1_ESM.pdf]

Supplementary Table 1. **Physical parameters of the martian environment and the low pressure environment chamber.** Laboratory measurements*, Diniega et al.[1], Cengel and Ghajar[2], Hech[3], Conway et al.[4], and Wagner and Pruß[5].

Variables	Mars	Low pressure environment chamber	Units	Description
g	3.75[5]	9.81[5]	$m\ s^{-2}$	gravitational constant
p	700*	700*	Pa	absolute pressure at experiment start
T_0	278 – 297*	278 – 297*	K	test bed surface temperature
λ	0.013[1]	0.26[1]	$W\ m^{-1}\ K^{-1}$	sand thermal conductivity
C_p	680[1]	830[1]	$J\ kg^{-1}\ K^{-1}$	sand heat capacity
k	1.0E-11[1]	1.3E-10[6]	m^2	sand permeability
ρ_s	1600[1]	1680[6]	$kg\ m^{-3}$	sand density
ρ_{ws}	1999[6]	1999[6]	$kg\ m^{-3}$	wet sand density
T_e	275[7]	275[7]	K	liquid water evaporation temperature
E_v	2.5E+6[4]	2.5E+6[4]	$J\ kg^{-1}$	enthalpy of evaporation of water
v	1.0E-5[4]	1.0E-5[4]	Pa s	gas viscosity
ρ_g	5.5E-3[4]	5.5E-3[4]	$kg\ m^{-3}$	gas density
θ	25*	25*	°	slope angle

Supplementary Table 2. Photogrammetry errors. Root Mean Square (RMS) errors [in mm] of source values and estimated values, and Reprojection Errors (RE) [in pix] calculated by Agisoft PhotoScan.

Marker	'cold' experiments						'warm' experiments					
	RUN 1		RUN 2		RUN 3		RUN 4		RUN 5		RUN 6	
	RMS	RE	RMS	RE	RMS	RE	RMS	RE	RMS	RE	RMS	RE
**1**	1.23	1.09	0.68	0.26	1.03	1.13	0.93	4.74	1.14	4.89	0.45	1.21
**2**	2.35	1.07	2.49	0.31	2.81	0.88	1.99	4.23	1.84	1.83	2.38	0.62
**3**	1.19	0.81	1.69	0.27	3.27	4.19	1.26	3.06	1.61	2.62	1.64	1.48
**4**	3.46	0.95	2.55	0.29	3.79	5.01	2.25	3.24	2.4	2.27	2.78	1.79
**5**	1.26	0.98	0.86	0.37	2.09	1.24	1.24	3.32	1.25	2.43	1.00	0.74
**6**	0.84	0.54	0.84	0.38	2.43	0.87	0.93	2.86	0.90	2.97	0.78	1.25
**7**	0.95	0.81	0.51	0.34	3.3	0.44	0.62	3.75	0.76	5.68	0.42	1.62
**8**	1.71	0.93	2.18	0.27	5.3	2.28	1.78	3.04	2.39	4.61	2.16	1.04
**9**	1.46	0.94	1.92	0.40	1.13	1.11	1.21	4.11	1.30	2.50	1.63	1.77
**10**	1.59	0.84	0.43	0.27	1.53	1.31	0.93	2.67	0.95	2.78	0.73	1.97
**11**	1.54	0.69	3.15	0.37	2.41	0.71	2.45	4.17	2.65	1.17	3.22	0.94
**12**	1.74	1.27	0.38	0.31	1.3	0.70	1.25	7.30	0.47	4.80	0.46	0.68

Supplementary Table 3. Errors for each transport-type of each experimental run. The 'Total error for Runs' is calculated by scaling the 'Measurement Error' to the total flow area. 'Interpolation Error' and 'Superposition Error' only apply for the subdivision of volumes into different transport-types (**Figure 1, Table 1**). The errors reported for each transport type in **Table 1**, are the largest of 'Measurement', 'Superposition' and 'Interpolation' errors reported here. 'MEAN' values were calculated using the propagation of errors.

	Run	Transport-types	'Interpolation Error' (cm^3)	'Superposition Error' (cm^3)	'Measurement Error' (cm^3)	Total error for Runs (cm^3)
'cold' experiments	**1**	Overland flows	-	-	7.6	**27.1**
		Percolation	-	-	18.4	
		Pellets	-	-	1.0	
		Dry avalanches/saltation	-	-	-	
	**2**	Overland flows	-	-	6.6	**20.3**
		Percolation	-	-	12.8	
		Pellets	-	-	0.9	
		Dry avalanches/saltation	-	-	-	
	**3**	Overland flows	-	-	3.2	**7.1**
		Percolation	-	-	3.7	
		Pellets	-	-	0.2	
		Dry avalanches/saltation	-	-	-	
	**MEAN**					**34.6**
'warm' experiments	**4**	Overland flows	3.4	-	2.2	**25.5**
		Percolation	-	-	8.4	
		Pellets	-	15.2	0.6	
		Dry avalanches/saltation	-	15.2	14.3	
	**5**	Overland flows	3.1	-	0.6	**9.0**
		Percolation	-	-	2.1	
		Pellets	-	14.4	0.9	
		Dry avalanches/saltation	-	14.4	5.4	
	**6**	Overland flows	3.2	-	1.4	**21.7**
		Percolation	-	-	5.4	
		Pellets	-	14.0	2.0	
		Dry avalanches/saltation	-	14.0	12.9	
	**MEAN**					**34.7**

Supplementary References

1. Diniega, S. *et al*. A new dry hypothesis for the formation of martian linear gullies. *Icarus* **225,** 526–537 (2013).
2. Cengel, Y. A. & Ghajar, A. J. Heat And Mass Transfer: Fundamentals And Applications. McGraw-Hill Education, New York, 992 p. (2014).
3. Hecht, M. H. Metastability of Liquid Water on Mars. *Icarus* **156,** 373–386 (2002).
4. Conway, S. J., Lamb, M. P., Balme, M. R., Towner, M. C. & Murray, J. B. Enhanced runout and erosion by overland flow at low pressure and sub-freezing conditions: Experiments and application to Mars. *Icarus* **211,** 443–457 (2011).

5. Wagner, W. & Pruß, A. The IAPWS Formulation 1995 for the Thermodynamic Properties of Ordinary Water Substance for General and Scientific Use. *J. Phys. Chem. Ref. Data* **31,** 387–535.
