# Supplementary material for: Water induced sediment levitation enhances downslope transport on Mars
Source: Nat Commun. 2017 Oct 27;8:1151. doi: 10.1038/s41467-017-01213-z (PMC5658360; doi:10.1038/s41467-017-01213-z)
Supplement: Supplementary file 3 — Description of Additional Supplementary Files [file 41467_2017_1213_MOESM3_ESM.pdf]

Description of Additional Supplementary Files

File Name: Supplementary Movie 1
Description: Close-up of 'cold' experiment Run1 (Table 1) during the 60s of water flow recorded from the camera outside the Mars simulation chamber (25 fps). Within the first 5 seconds some single pellets are ejected at the contact of sediment and water and rolled down the slope. Sporadic appearance of bubbles at the surface of the flow show the water is boiling. See also Figure 1 a-c for detailed image, map, and elevation data of the endstate of this experiment.

File Name: Supplementary Movie 2
Description: 'Cold' experiment Run3 (Table 1) during the 60s of water flow recorded from a webcam inside the Mars simulation chamber (30 fps). Only one larger pellet is ejected and rolls down the slope (00:00:04:00). Movement of sediment stops immediately after the water flow is stopped (00:01:03:00).

File Name: Supplementary Movie 3
Description: 'Warm' experiment Run5 (Table 1) during 60s of water flow and ~30 s afterwards showing ongoing grain ejection and dry avalanches. Recorded using a camera outside the Mars simulation chamber (30 fps). Note the short-lived transportation "channel" from 00:00:03:00 to 00:00:09:00, which is completely filled with material in the form of pellets and dry avalanches. Water introduction is stopped at 00:01:01:00, but water in the sediment continues to boil which triggers further transportation. See also Figure 1 d-f for detailed image, map, and elevation data of the end-state and Figure 3 for a detailed description of the behaviour of the flow at different times.

File Name: Supplementary Movie 4
Description: 'Warm' experiment Run5 (Table 1) during the 60s of water flow and continuing for ~60 s afterwards, showing the last detectable grain ejection. Recorded from a webcam inside the Mars simulation chamber (14 fps). This movie shows the same experiment as presented in Supplementary Movies 3 and 6, but with a focus on the region where the water was introduced. See also Figure 1 d-f for detailed image, map, and elevation data of the end-state and Figure 3 for a detailed description of the behaviour of the flow at different times.

File Name: Supplementary Movie 5
Description: High speed camera (1000 fps) recording of 'warm' experiment Run4 (Table 1) of ejecting sand grains at the contact between wet sediment (flow front) and dry sediment.

File Name: Supplementary Movie 6
Description: 'Warm' experiment Run5 (Table 1) during water flow and ~30 s afterwards showing dry avalanches. This movie shows the same experiment presented in Supplementary Movies 3 and 4, but with a focus on the transportation "channel" (between 00:00:03:00 and 00:00:09:00) and dry avalanches (after ~00:00:28:00). Dry avalanches superpose pellets and areas eroded by pellet levitation. Recorded from a webcam inside the Mars simulation chamber with a top-down view (20 fps). See also Figure 1 d-f for detailed image, map, and elevation data of the end-state and Figure 3 for a detailed description of the behaviour of the flow at different times.
